# Biocompatibility and Comfort during Extended Wear of Mel4 Peptide-Coated Antimicrobial Contact Lenses

**DOI:** 10.3390/antibiotics11010058

**Published:** 2022-01-03

**Authors:** Parthasarathi Kalaiselvan, Debarun Dutta, Nagaraju Konda, Pravin Krishna Vaddavalli, Savitri Sharma, Fiona Stapleton, Mark D. P. Willcox

**Affiliations:** 1 School of Optometry and Vision Science, UNSW Sydney, Sydney, NSW 2041, Australia; d.dutta@aston.ac.uk (D.D.); f.stapleton@unsw.edu.au (F.S.); m.willcox@unsw.edu.au (M.D.P.W.); 2 School of Optometry, Aston University, Birmingham B4 7ET, UK; 3 School of Medical Sciences, University of Hyderabad, Hyderabad 500 046, India; knr@uohyd.ac.in; 4 Bausch & Lomb Contact Lens Centre, L V Prasad Eye Institute, Hyderabad 500 034, India; pravin@lvpei.org; 5 The Cornea Institute, L V Prasad Eye Institute, Hyderabad 500 034, India; 6 Jhaveri Microbiology Centre, L V Prasad Eye Institute, Hyderabad 500 034, India; savitri@lvpei.org

**Keywords:** Mel4 peptide, antimicrobial contact lens, extended wear, biocompatibility, comfort, clinical trail

## Abstract

(1) Purpose: This study aimed to investigate the effects of Mel4 antimicrobial contact lenses (MACL) on the ocular surface and comfort during extended wear. (2) Methods: A prospective, randomised, double-masked, contralateral clinical trial was conducted with 176 subjects to evaluate the biocompatibility of contralateral wear of MACL. The wearing modality was 14-day extended lens wear for three months. The participants were assessed at lens dispensing, after one night, two weeks, one month and three months of extended wear and one month after study completion. (3) Results: There were no significant differences (*p* > 0.05) in ocular redness or palpebral roughness between Mel4 and control eyes at any of the study visits. There was no significant difference (*p* > 0.05) in corneal staining between Mel4 and control eyes. There were no significant differences in front surface wettability or deposits or back surface debris (*p* > 0.05). No statistically significant differences (*p* > 0.05) were found in comfort, dryness, CLDEQ-8 scores lens or edge awareness. There was no evidence for delayed reactions on the ocular surface after cessation of lens wear. (4) Conclusion: The novel MACLs showed similar comfort to control lenses and were biocompatible during extended wear. Thus, these lenses were compatible with the ocular surface.

## 1. Introduction

Contact lens wear can be associated with inflammatory and infective responses, triggered by microbial colonisation of contact lenses. These are major concerns for contact lens wearers and practitioners. The development of contact lenses with antimicrobial activity may inhibit microbial adhesion and so reduce contact-lens-related inflammation and infection. Several antimicrobial contact lenses have been developed and tested in laboratory models. These include contact lenses containing silver [1,2,3], inhibitors of bacteria quorum-sensing systems [4], poly-epsilon lysine [5] and nitric-oxide-releasing lenses [6]. A cationic, peptide-coated (melimine) contact lens has also been developed that showed good antimicrobial activity in vitro [7], prevented bacterially-driven adverse events associated with contact lens wear in animal models [8,9] and was generally safe to wear in humans, although it was associated in some wearers with low levels of corneal staining [10]. Due to this latter issue, the cationic peptide melimine was shortened to make Mel4 [11].

Mel4, a small, cationic, antimicrobial peptide, has high antimicrobial activity against *Pseudomonas aeruginosa* and *Staphylococcus aureus* in solution and when immobilised on surfaces [12]. It has been successfully coated onto hydrogel and silicone hydrogel contact lenses [12,13,14] and shown to be active against other bacteria such as *Stenotrophomonas maltophilia* and *Delftia acidovorans*. The Mel4-coated lenses were safe in a rabbit model of daily contralateral wear [13,14]. In addition, a phase I, human clinical trial showed no corneal fluorescein staining and no increase in ocular redness after one week of daily wear [13].

A phase II/III clinical trial on the Mel4-coated contact lenses was conducted at the LV Prasad Eye Institute in Hyderabad, India, and the biocompatibility of the lenses is addressed in the current manuscript. The main aim of this trial was to assess whether the Mel4-coated lenses could reduce the incidence of corneal inflammatory events during extended wear. These lenses resulted in a reduction in the incidence of corneal inflammatory events by 69% [15]. These Mel4-coated lenses had similar levels and types of microbes isolated from them and from eyes wearing them compared to the control lenses [16].

It is also valuable to investigate the biocompatibility and comfort of Mel4-coated contact lenses on the extended wear modality. Thus, the aim of the current study was to investigate the biocompatibility and comfort of Mel4-coated contact lenses during the phase II/III, extended wear, human clinical trial. The hypotheses of this study were that the Mel4-coated contact lenses are compatible and comfortable during extended wear.

## 2. Results

### 2.1. Assessment of Activity of Mel4-Coated Lenses Prior to Lens Wear

The data for the amount of Mel4 on contact lenses and the ability of lenses to inhibit the adhesion of *Pseudomonas aeruginosa* and *Staphylococcus aureus* have been previously published [15]. Briefly, randomly selected contact lenses from each batch that was produced were selected for measurement. These Mel4-coated lenses contained 62.6 ± 26.4 µg of amino acids per lens and significantly reduced the adhesion of *P. aeruginosa* and *S. aureus* by >1.8 log10 CFU/lens (*p* < 0.001) compared to control uncoated lenses [15]. This demonstrated that the participants in the trial were prescribed with active Mel4-coated contact lenses.

### 2.2. Subject Demographics

The demographic and biometric data for the subjects who were dispensed with study lenses are summarised in Table 1. Slightly more males (108/208; 52%) were enrolled and were dispensed with study lenses (93/176; 53%) than females. Additionally, a greater number of neophytes (160/208; 77%) were enrolled and were dispensed with study lenses (128/176; 73%). There was no difference in the refractive errors, keratometry and contact lens powers between the Mel4- and control-lens-wearing eyes in both enrolled (*p* > 0.05) and study-lens-dispensed subjects (*p* > 0.05).

### 2.3. Clinical Lens Surface Characteristics

The lens surface characteristics of Mel4 and control contact lenses are presented in Table 2. There were no significant differences in front surface wettability between Mel4 and control lenses during all the visits (*p* > 0.05), with values ranging from 3.7 to 3.5 units. The front surface wetting for both Mel4 and control lenses decreased by 0.1–0.2 units over the course of the study, and this was significant (*p* = 0.001). There were no significant differences seen either in front surface deposits (*p* > 0.05) or back surface debris (*p* > 0.05) across all the study visits between both lens types. The front surface deposits for both lens types increased over the course of the study by between 0.1 and 0.5 units (*p* = 0.001), and the back surface debris significantly increased over the course of the study by between 0.1 and 0.3 units (*p* = 0.001).

### 2.4. Lens Fit Characteristics

The lens fit characteristics of Mel4 and control contact lenses are presented in Table 3. There were no significant differences in any lens fit characteristics between Mel4 and control contact lenses over the course of the study. The average overall lens acceptance score for both the lens types at each visit was 3.8 which indicated good centration, complete coverage, acceptable tightness of the lens and adequate lens movement and lens lag. No lens was refitted during the study period because of any lens fit issues. There were small but statistically significant differences between the visits for primary gaze movement, lag, tightness and overall acceptance (*p* < 0.05). There was no significant difference between the visits for lens centration (*p* ≥ 0.05). No mucin balls were seen with either of the lens types at any of the visits.

### 2.5. Ocular Physiology

The conjunctival redness and roughness of the Mel4 and control lens wearing eye at the 1N, 2W, 1M and 3M study visits are presented in Table 4. There was no significant difference in bulbar redness (*p* > 0.7), limbal redness (*p* > 0.9), palpebral redness (*p* > 0.6) or palpebral roughness (*p* > 0.3) between Mel4- and control-contact-lens-wearing eyes in any of the study visits. All of these variables slightly but significantly (*p* = 0.001) increased by between 0.1 and 0.2 units over the course of the study. Similarly, there were no significant differences in lens-induced conjunctival staining (*p* = 1.0) or indentation (*p* > 0.1) between the Mel4-lens-wearing eye and control-lens-wearing eye (Table 4). There were no significant differences (*p* > 0.35) in central, nasal, temporal or superior corneal staining (extent, depth or type) between Mel4- and control-contact-lens-wearing eyes (Table 5). None of these corneal-staining characteristics changed during the study (*p* ≥ 0.2).

### 2.6. Subjective Ratings

The subjective ratings of the comfort at each visit are presented in Table 6. There was no significant difference (*p* > 0.1) in the subjective ratings of overall comfort, dryness or lens edge awareness between Mel4- and control-lens-wearing eyes. Overall comfort significantly (*p* = 0.001) decreased for both lens types from the 1N to the 3M visits, dropping by 3–4 points, as did overall dryness (*p* = 0.001) which increased by 5 points.

**Figure 1:**
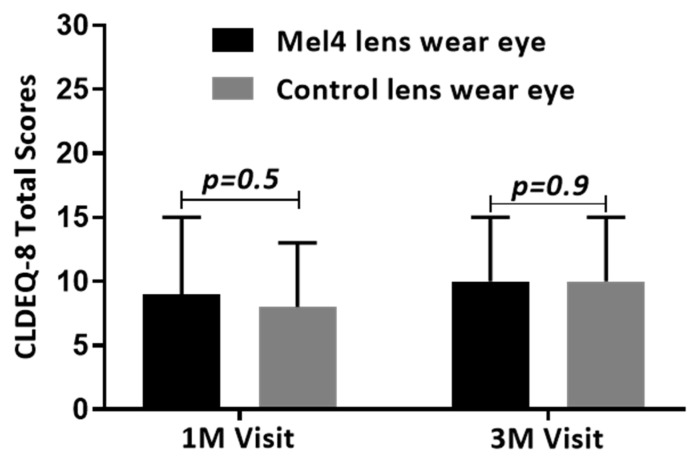
Comfort of contact lenses during wear measured with the modified CLDEQ-8.

All subjects were asked to score the modified CLDEQ-8 questionnaires at the one-month and three-month visit. One hundred and thirty-nine subjects scored the questionnaires at the first month’s visit and 126 subjects scored the questionnaires at the three-month visit. There were no significant differences in CLDEQ-8 scores between Mel4- and control-lens-wearing eyes at either the one- (*p* = 0.5) or three-month visit (*p* = 0.9) (Figure 1).

### 2.7. Ocular Responses One Month after Cessation of Lens Wear

The ocular responses at the four-month visit, i.e., one month after cessation of lens wear, are provided in Supplementary Table S2. There were no statistical differences between the Mel4-lens-wearing eyes and the control-lens-wearing eyes for any of the clinical or subjective variables. There were some small but significant differences between the study visits for conjunctival bulbar, limbal and palpebral redness (*p* = 0.001), which had increased by 0.1 to 0.2 units, and palpebral roughness (*p* = 0.001), which had increased by 0.1 unit. There was also a very small but significant change in inferior corneal staining (extent, depth and type; *p* ≤ 0.016), which had increased by less than a unit interval. Overall, ocular comfort was slightly but significantly (*p* = 0.001) decreased at the four-month visit by 3 units. As each of these differences were not different between lenses and there were no lens/visit interactions in the statistical analyses, these changes were likely the result of lens wear rather than the result of wearing either Mel4-coated or control lenses.

### 2.8. Ocular Responses of Participants Who Dropped out of Lens Wear during the Study Compared to Those Who Completed the Study

The ocular and subjective responses of the participants who dropped out of lens wear during the study compared to those who completed the study at different study visits are provided in Supplementary Table S3. The responses collected from four participants at the 1N visit that had dropped out by the 2W visit were compared to the participants who remained wearing lenses at the 2W visit (153). Similarly, two participants at the 2W visit that had dropped out by the 1M visit and their responses were compared to the participants who remained wearing lenses at the 1M visit (144), and two participants at the 1M visit that had dropped out by the 3M visit were compared to the participants who remained wearing lenses at the 3M visit (129). There were no clinical differences in ocular and subjective responses between dropouts and those who remained in Mel4 or control lenses. The lens surface characteristics and lens fit characteristics of the participants who dropped out of lens wearing during the study compared to those who completed the study at different study visits are provided in Supplementary Table S4. There were no clinical differences in lens surface characteristics and lens fit characteristics between dropouts and those who remained in Mel4 or control lenses.

## 3. Discussion

This study investigated the biocompatibility and comfort of Mel4-coated contact lenses in a human, three-month, extended wear clinical trial. Overall, in comparison to the eyes wearing control, uncoated, etafilcon A lenses, Mel4-coated lenses had no worse effect on the biocompatibility and comfort during lens wear than the uncoated lenses. This indicates that Mel4-coated lenses are not prone to forming more deposits, wet as well as control lenses, do not affect lens parameters that could influence centration, movement or tightness and do not induce inflammation as measured by changes in redness or conjunctival or corneal staining or comfort during lens wear. This is in agreement with a one-week study of daily wear with Mel4-coated lenses [13] and is similar to results reported for other antimicrobial lenses that had been made using a fimbrolide [4] or another cationic, antimicrobial peptide (a forerunner of Mel4), melimine [10]. Wearing Mel4-coated contact lenses over a three-month period did not result in any delayed ocular reactions as the data from the 4M visit, at which time the participants had been not wearing Mel4-coated lenses for one month (but had returned to their habitual method for correcting their refractive error), were not different between the Mel4- or control-lens-wearing eyes.

This study found no significant fluorescein staining (extent, depth and type) of the cornea following lens wear of Mel4-coated lenses, which is similar to the study on Mel4-coated lenses worn on a daily-wear basis for a week [13] and fimbrolide-coated lenses after 20 to 22 h of lens wear [4]. However, this does contrast with a study of melimine-coated contact lenses which were associated with corneal staining [10]. The current study confirms that the change in amino acid sequence between melimine (TLISWIKNKRKQRPRVSRRRRRRGGRRRR) and Mel4 (KNKRKRRRRRRGGRRRR) eliminated the corneal punctate staining that occurred with melimine-coated lenses. The change from melimine to Mel4 removed several amino acids. Tryptophan (W) is present in the amino acid sequence of melimine at position five. Also present in melimine, are other hydrophobic amino acids such as isoleucine (I, two residues at positions 3 and 6), glycine (G, two residues at positions 24 and 25), valine (V, one residue at position 16), leucine (L, one residue at position 2) and proline (P, one residue at position 14). None of these amino acids are in Mel4. It is possible that one or more of these might have been involved in interactions with human corneal cells. The amino acid tryptophan (W) is often present in proteins that reside within membranes [17,18] which indicates its potential to interact with membranes. Additionally, tryptophan in arginine-rich peptides facilitates the translocation of these peptides through membranes [19].

Similar to melimine coating [7], the Mel4 coating (22.7° ± 5.0) has previously been shown to significantly improve the wettability of etafilcon A lenses (69.3° ± 14.6) when measured using the advancing contact angle technique in the laboratory [13]. This reinforces previous findings that wettability measured in the laboratory does not translate to improved comfort responses [20] as there was no difference in comfort between Mel4-coated and control lenses. Fimbrolide-coated lenses [4] have been associated with increased dryness, lens edge and lens awareness and were slightly less comfortable to wear. In the current study, also there was no significant difference in dryness, lens edge and lens awareness between Mel4-coated and control lenses, similar to the study of Mel4-coated lenses [13] used on a daily-wear basis for a week or melimine-coated lenses [10] when worn for a day. The reason for the improved comfort response with Mel4-coated lenses compared to fimbrolide lenses may be due to the different antimicrobial compounds themselves, the different chemistries used to attach fimbrolide and Mel4 to lenses or the different lenses used (hydrogel etafilcon A vs. silicone hydrogel lotrafilcon A). 

To understand whether dropout from this clinical trial at any stage was associated with differences in the ocular surface or lens characteristics of the people who dropped out compared to those that completed the trial, variables of those who dropped out were compared to those who remained in the trial at each visit and at the final 3-month visit. This demonstrated that the people who dropped out did not have any significant differences in ocular surface responses, lens characteristics or comfort. This further reinforces the biocompatibility of Mel4 during lens wear.

## 4. Materials and Methods

### 4.1. Study Design and Participants

The study design, inclusion and exclusion criteria were identical to a previous study [16]. Briefly, a total of 176 participants who met the inclusion/exclusion criteria were dispensed with study lenses. Participants were randomly assigned to wear a Mel4 antimicrobial contact lens (MACL) in one eye and a control lens (uncoated etafilcon A) in the contralateral eye. All the participants were instructed to replace lenses every two weeks during three months of extended wear. To reduce the possibility of participants mixing right- and left-eye lenses, the right-eye and left-eye lens vials were affixed with green and white labels, respectively. If the participants needed to remove their lenses temporarily, they were given Biotrue contact lens care solution (Bausch and Lomb, Rochester, NY, USA) and a case only for temporary storage. 

### 4.2. Production and Quantification of Mel4 Peptide Attached to Contact Lenses

Etafilcon A contact lenses (Acuvue2^®^, Johnson and Johnson Vision Care Inc., Jacksonville, FL, USA) were used for this study. Mel4 peptide (amino acid sequence: KNKRKRRRRRRGGRRRR; American Peptide Company, Sunnyvale, CA, USA) was synthesised by conventional solid-phase peptide synthesis with >95% purity. The procedure for covalently attaching Mel4 to contact lenses has been reported elsewhere [10,13,16]. Control, uncoated (etafilcon A) lenses were removed from their packs, washed and autoclaved prior to use. Mel4-coated lenses for the clinical trial were produced in different batches. After the production of each batch and prior to the lens dispensing visit, two Mel4-coated lenses from each batch were assessed by amino acid analysis to confirm the presence and amount of peptide on to the lens surface [12,13,21]. The sum of all the amino acids derived from each contact lens was regarded as the total amount of peptide attachment to a contact lens. Similarly, two Mel4-coated and control lenses from each batch were assessed for adhesion of *P. aeruginosa* (ATCC 27853) and *S. aureus* (L2260/15). The bacterial adhesion protocol has been reported previously [7,12].

### 4.3. Clinical Procedures

A total of seven visits were undertaken; baseline (visit 1), lens dispensing (visit 2), after one night (visit 3), 2 weeks, (visit 4), 1 month (visit 5) and 3 months of lens wear (visit 6), followed by a 1-month follow-up visit after study lens discontinuation (visit 7). The seventh follow-up visit at the end of 3 months’ extended wear included no assigned contact lens wear, and the subjects were free to wear their glasses if desired to test for any delayed responses to the investigational product. At each scheduled visit, the ocular characteristics and subjective responses of each participant were assessed. Slit-lamp biomicroscopy was performed for anterior eye assessment including contact lens fit, lens surface characteristics, ocular redness (bulbar, limbal and palpebral), palpebral roughness, conjunctival and corneal staining. Contact lens fitting (assessed using the push-up test) and contact lens deposits during wear were measured according to previously described methods [22,23] All of the clinical grading was conducted using the Cornea and Contact Lens Research Unit grading scales [24] (0 to 4 units) interpolated into 0.1 increments, except for corneal staining which was graded in 1.0 steps for extent and depth and 0.5 steps for type. Concordance training for the optometrists was conducted before study commencement, and concordance was measured every 6 months during the study. All optometrists were allowed to examine study participants if they scored more than 70% concordance for each grading scale, and they were retrained if concordance dropped below this level. All optometrists were masked to which eye of each participant was wearing which contact lenses. The CLDEQ-8 questionnaire was modified for monocular lens wear (see Supplementary Table S1). 

### 4.4. Statistical Analysis

Data were analysed using Microsoft^®^ Office Excel^®^, Graph Pad Prism 7.02 (Graph Pad Software Inc., San Diego, CA, USA) and IBM SPSS (Package for the Social Sciences software) for Windows software v24.0 (SPSS, Inc., Chicago, IL, USA). According to central limit theorem, [25] this study, with its large sample collection of ordinal variables, can be assumed to have a normal distribution; thus, the means of the variables were reported with standard deviation. Non-ordinal variables were described as median and range. The comparisons of clinical and lens variables at each visit between Mel4- and control-lens-wearing eyes were examined using linear mixed models (LMM). As some subjects dropped out of lens wear during the study and there were some other cases of missing data, the sample size varied among the study visits and so LMM analyses were conducted. Post hoc *t*-tests with Bonferroni correction were used to assess significant changes over time and between both lens-wear types. For the subjects who dropped out of the study, where data had been collected, this was compared to the subjects who remained in the study to determine whether changes to ocular surface physiology or comfort during wear may have influenced the decision to drop out of the study. For all tests, the level of statistical significance was maintained at *p* < 0.05.

## 5. Conclusions

In conclusion, wearing Mel4-coated contact lenses did not affect the ocular responses, contact lens deposition or other characteristics or comfort during lens wear. These data, combined with the fact that the Mel4-coated lenses did not affect the normal ocular microbiota during wear [16] and could reduce the number of corneal infiltrative events during extended contact lens wear [15], indicate that Mel4 coatings are biocompatible and useful to control microbially-driven adverse events.

## Figures and Tables

**Table 1:** Demographic and biometric data of subjects dispensed with study lenses.

Demographic and Biometric Details	Subjects Dispensed with Study Lenses (*n* = 176)		
	Mel4-Lens-Wearing Eye	Control-Lens-Wearing Eye	*p*-Value
Age (years): Mean ± SD Range	22.6 ± 4.2 18 to 42		-
Gender (Male:Female)	93:83		-
Refractive error-Sphere (Ds) ^1^: Mean ± SD; Range	−2.82 ± 1.44 −0.50 to −6.50	−2.80 ± 1.46 −0.50 to −6.50	0.528
Refractive error-Cylinder (Dc): Mean ± SD; Range	−0.25 ± 0.35 −0.25 to −1.50	−0.22 ± 0.35 −0.25 to −1.50	0.249
Keratometry-Flat (D): Mean ± SD; Range	43.02 ± 1.44 37.50 to 47.25	43.01 ± 1.46 37.50 to 47.25	0.663
Keratometry-Steep (D): Mean ± SD; Range	43.76 ± 1.58 38.75 to 48.50	43.71 ± 1.58 38.75 to 48.50	0.105
Contact lens wearer (Neophyte:Experienced lens wearer)	128:48		-
Contact lens base curve (8.3:8.7) mm	106:70		-
Contact lens power (Ds): Mean ± SD; Range	−2.84 ± 1.36 −1.00 to −6.00	−2.84 ± 1.39 −1.00 to −6.00	0.869

^1^ D = diopter.

**Table 2:** Surface characteristics of Mel4 and control contact lenses at various study visits.

Variables (Range, Incremental Steps)	Visits with Lens *	Number of Samples	Mel4 Lens	Control Lens	Linear Mixed Model		
			(Mean ± SD)	(Mean ± SD)	Lens (Mel4 vs. Control)	Visit	Lens vs. Visit
Front surface wetting (0–4, 0.1)	Lens Dispensing	176	3.7 ± 0.6	3.7 ± 0.6	0.593	0.001	0.513
	1N	165	3.6 ± 0.6	3.7 ± 0.5			
	2W	153	3.6 ± 0.3	3.6 ± 0.3			
	1M	144	3.6 ± 0.3	3.5 ± 0.3			
	3M	128	3.6 ± 0.3	3.5 ± 0.3			
Front surface deposits (0–4, 0.1)	Lens Dispensing	176	0.2 ± 0.3	0.2 ± 0.3	0.896	0.001	0.996
	1N	165	0.3 ± 0.4	0.3 ± 0.4			
	2W	153	0.6 ± 0.5	0.6 ± 0.5			
	1M	144	0.6 ± 0.6	0.6 ± 0.6			
	3M	128	0.7 ± 0.6	0.7 ± 0.6			
Back surface debris (0–4, 0.1)	Lens Dispensing	176	0.1 ± 0.2	0.1 ± 0.2	0.715	0.001	0.857
	1N	165	0.2 ± 0.2	0.2 ± 0.2			
	2W	153	0.3 ± 0.4	0.3 ± 0.4			
	1M	144	0.4 ± 0.5	0.4 ± 0.5			
	3M	128	0.4 ± 0.5	0.4 ± 0.5			

* 1N = 1 night of lens wear, 2W = 2 weeks of lens wear, 1M = 1 month on lens wear, 3M = 3 months of lens wear.

**Table 3:** Lens fit characteristics of Mel4 and control contact lenses at various study visits.

Variables (Range, Incremental Steps)	Visits with Lens *	Number of Samples	Mel4 Lens	Control Lens	Linear Mixed Model		
			(Mean ± SD)/ (Median and Range)	(Mean ± SD)/ (Median and Range)	Lens	Visit	Lens vs. Visit
Centration X-axis (−1 to +1, 0.1 mm)	Lens Dispensing	176	0 (−0.3–0.2)	0 (−0.3–0.2)	0.589	0.409	0.950
	1N	165	0 (−0.5–0.3)	0 (−0.5–0.3)			
	2W	153	0 (−0.3–0.2)	0 (−0.3–0.2)			
	1M	144	0 (−0.5–0.2)	0 (−0.5–0.0)			
	3M	128	0 (−0.3–0.2)	0 (−0.3–0.2)			
Centration Y-axis (−1 to +1, 0.1 mm)	Lens Dispensing	176	0 (−0.3–0.5)	0 (−0.3–0.3)	0.595	0.118	0.266
	1N	165	0 (−0.4–0.5)	0 (−0.3–0.5)			
	2W	153	0 (−0.3–0.5)	0 (−0.3–0.5)			
	1M	144	0 (−0.4–0.4)	0 (−0.3–0.4)			
	3M	128	0 (−0.2–0.3)	0 (−0.2–0.3)			
Primary gaze movement (0–10, 0.1)	Lens Dispensing	176	0.4 ± 0.1	0.4 ± 0.1	0.988	0.049	0.752
	1N	165	0.4 ± 0.1	0.4 ± 0.1			
	2W	153	0.4 ± 0.1	0.4 ± 0.1			
	1M	144	0.4 ± 0.1	0.4 ± 0.1			
	3M	128	0.4 ± 0.1	0.4 ± 0.1			
Primary gaze lag (0–10, 0.1)	Lens Dispensing	176	0.1 ± 0.1	0.1 ± 0.1	1.000	0.005	-
	1N	165	0.1 ± 0.1	0.1 ± 0.1			
	2W	153	0.1 ± 0.1	0.1 ± 0.1			
	1M	144	0.2 ± 0.1	0.2 ± 0.1			
	3M	128	0.2 ± 0.1	0.2 ± 0.1			
Tightness (0–100, 1)%	Lens Dispensing	176	41 ± 3	41 ± 3	0.817	0.001	0.968
	1N	165	41 ± 3	41 ± 3			
	2W	153	42 ± 3	42 ± 3			
	1M	144	41 ± 3	41 ± 3			
	3M	128	42 ± 3	42 ± 3			
Overall acceptance (0–4, 0.1)	Lens Dispensing	176	3.8 ± 0.1	3.8 ± 0.1	0.410	0.001	0.380
	1N	165	3.8 ± 0.1	3.8 ± 0.1			
	2W	153	3.8 ± 0.1	3.8 ± 0.1			
	1M	144	3.8 ± 0.1	3.8 ± 0.1			
	3M	128	3.8 ± 0.1	3.8 ± 0.1			

* 1N = 1 night of lens wear, 2W = 2 weeks of lens wear, 1M = 1 month on lens wear, 3M = 3 months of lens wear.

**Table 4:** Conjunctival responses during contact lens wear.

Variables (Range, Incremental Steps)	Visits with Lens *	Number of Samples	Mel4 Lens	Control Lens	Linear Mixed Model		
			(Mean ± SD)	(Mean ± SD)	Lens (Mel4 vs. Control)	Visit	Lens vs. Visit
Bulbar Redness (0–4, 0.1)	1N	167	1.5 ± 0.2	1.5 ± 0.2	0.758	0.001	0.135
	2W	153	1.5 ± 0.2	1.5 ± 0.2			
	1M	144	1.6 ± 0.2	1.6 ± 0.2			
	3M	129	1.6 ± 0.2	1.6 ± 0.2			
Limbal Redness (0–4, 0.1)	1N	167	1.2 ± 0.3	1.2 ± 0.3	0.961	0.001	0.660
	2W	153	1.2 ± 0.2	1.2 ± 0.2			
	1M	144	1.3 ± 0.3	1.3 ± 0.3			
	3M	129	1.4 ± 0.2	1.4 ± 0.2			
Palpebral Redness (0–4, 0.1)	1N	167	1.5 ± 0.2	1.6 ± 0.2	0.610	0.001	0.053
	2W	153	1.6 ± 0.3	1.6 ± 0.3			
	1M	144	1.6 ± 0.3	1.6 ± 0.3			
	3M	129	1.7 ± 0.3	1.7 ± 0.3			
Palpebral Roughness (0–4, 0.1)	1N	167	1.2 ± 0.3	1.3 ± 0.3	0.388	0.001	0.574
	2W	153	1.3 ± 0.3	1.3 ± 0.3			
	1M	144	1.3 ± 0.3	1.3 ± 0.3			
	3M	129	1.4 ± 0.3	1.4 ± 0.3			
Lens Induced Conjunctival Staining (0–4, 0.1)	1N	167	0.2 ± 0.2	0.2 ± 0.2	1.000	0.001	1.000
	2W	153	0.2 ± 0.3	0.2 ± 0.2			
	1M	144	0.3 ± 0.3	0.3 ± 0.3			
	3M	129	0.3 ± 0.3	0.3 ± 0.3			
Lens Induced Conjunctival Indentation (0–4, 0.1)	1N	167	0.1 ± 0.1	0.1 ± 0.1	0.112	0.001	0.041
	2W	153	0.1 ± 0.2	0.1 ± 0.2			
	1M	144	0.1 ± 0.2	0.1 ± 0.2			
	3M	129	0.1 ± 0.2	0.1 ± 0.2			

* 1N = 1 night of lens wear, 2W = 2 weeks of lens wear, 1M = 1 month on lens wear, 3M = 3 months of lens wear.

**Table 5:** Corneal staining during contact lens wear.

Variables (Type; Range, Incremental Steps)	Visits with Lens *	Number of Samples	Mel4 Lens	Control Lens	Linear Mixed Model		
			(Median and Range)	(Median and Range)	Lens (Mel4 vs. Control)	Visit	Lens vs. Visit
Centre (Extent; 0–4, 1)	1N	162	0 (0–0)	0 (0–0)	0.674	0.200	0.632
	2W	153	0 (0–1)	0 (0–1)			
	1M	144	0 (0–0)	0 (0–1)			
	3M	129	0 (0–1)	0 (0–1)			
Centre (Depth; 0–4, 1)	1N	162	0 (0–0)	0 (0–0)	0.674	0.200	0.632
	2W	153	0 (0–1)	0 (0–1)			
	1M	144	0 (0–0)	0 (0–1)			
	3M	129	0 (0–1)	0 (0–1)			
Centre (Type; 0–4, 0.5)	1N	162 *	0 (0–0)	0 (0–0)	0.674	0.200	0.632
	2W	153	0 (0–1)	0 (0–1)			
	1M	144	0 (0–0)	0 (0–1)			
	3M	129	0 (0–1)	0 (0–1)			
Nasal (Extent; 0–4, 1)	1N	162	0 (0–1)	0 (0–1)	0.670	0.426	0.262
	2W	153	0 (0–1)	0 (0–1)			
	1M	144	0 (0–1)	0 (0–2)			
	3M	129	0 (0–0)	0 (0–1)			
Nasal (Depth; 0–4, 1)	1N	162	0 (0–1)	0 (0–1)	0.869	0.421	0.254
	2W	153	0 (0–1)	0 (0–1)			
	1M	144	0 (0–1)	0 (0–2)			
	3M	129	0 (0–0)	0 (0–1)			
Nasal (Type; 0–4, 0.5)	1N	162	0 (0–1)	0 (0–1.5)	0.586	0.386	0.342
	2W	153	0 (0–1)	0 (0–1)			
	1M	144	0 (0–1)	0 (0–2)			
	3M	129	0 (0–0)	0 (0–1)			
Temporal (Extent; 0–4, 1)	1N	162	0 (0–1)	0 (0–1)	0.856	0.437	0.457
	2W	153	0 (0–1)	0 (0–1)			
	1M	144	0 (0–0)	0 (0–1)			
	3M	129	0 (0–1)	0 (0–0)			
Temporal (Depth; 0–4, 1)	1N	162	0 (0–1)	0 (0–1)	0.856	0.437	0.457
	2W	153	0 (0–1)	0 (0–1)			
	1M	144	0 (0–0)	0 (0–1)			
	3M	129	0 (0–1)	0 (0–0)			
Temporal (Type; 0–4, 0.5)	1N	162	0 (0–1)	0 (0–1)	0.780	0.340	0.498
	2W	153	0 (0–1)	0 (0–1)			
	1M	144	0 (0–0)	0 (0–0.5)			
	3M	129	0 (0–1)	0 (0–0)			
Superior (Extent; 0–4, 1)	1N	162	0 (0–0)	0 (0–1)	0.368	0.242	0.362
	2W	153	0 (0–1)	0 (0–1)			
	1M	144	0 (0–1)	0 (0–2)			
	3M	129	0 (0–1)	0 (0–1)			
Superior (Depth; 0–4, 1)	1N	162	0 (0–0)	0 (0–1)	0.368	0.242	0.362
	2W	153	0 (0–1)	0 (0–1)			
	1M	144	0 (0–1)	0 (0–2)			
	3M	129	0 (0–1)	0 (0–1)			
Superior (Type; 0–4, 0.5)	1N	162	0 (0–0)	0 (0–1)	0.649	0.151	0.258
	2W	153	0 (0–1)	0 (0–0.5)			
	1M	144	0 (0–1)	0 (0–1.5)			
	3M	129	0 (0–1)	0 (0–1)			
Inferior (Extent; 0–4, 1)	1N	162	0 (0–1)	0 (0–1)	0.119	0.238	0.337
	2W	153	0 (0–1)	0 (0–2)			
	1M	144	0 (0–1)	0 (0–2)			
	3M	129	0 (0–2)	0 (0–2)			
Inferior (Depth; 0–4, 1)	1N	162	0 (0–1)	0 (0–1)	0.119	0.238	0.337
	2W	153	0 (0–1)	0 (0–2)			
	1M	144	0 (0–1)	0 (0–2)			
	3M	129	0 (0–2)	0 (0–2)			
Inferior (Type; 0–4, 0.5)	1N	162	0 (0–1)	0 (0–1)	0.119	0.238	0.337
	2W	153	0 (0–1)	0 (0–2)			
	1M	144	0 (0–1)	0 (0–2)			
	3M	129	0 (0–2)	0 (0–2)			

* 1N = 1 night of lens wear, 2W = 2 weeks of lens wear, 1M = 1 month on lens wear, 3M = 3 months of lens wear.

**Table 6:** Ocular comfort responses during contact lens wear.

Variables (Range, Incremental Steps)	Visits with Lens *	Number of Samples	Mel4 Lens	Control Lens	Linear Mixed Model		
			(Mean ± SD)	(Mean ± SD)	Lens (Mel4 vs. Control)	Visit	Lens vs. Visit
Overall comfort (1–100, 1)	1N	167	92 ± 8	92 ± 8	0.770	0.001	0.556
	2W	153	91 ± 7	91 ± 8			
	1M	144	90 ± 7	91 ± 9			
	3M	129	88 ± 8	89 ± 7			
Overall dryness (1–100, 1)	1N	167	8 ± 5	8 ± 5	0.789	0.001	0.742
	2W	153	11 ± 11	11 ± 11			
	1M	144	11 ± 11	12 ± 12			
	3M	129	13 ± 11	13 ± 11			
Edge awareness (0–10, 1)	1N	165	1 (1–2)	1 (1–2)	0.135	0.077	0.969
	2W	153	1 (1–4)	1 (1–5)			
	1M	144	1 (1–3)	1 (1–3)			
	3M	128	1 (1–3)	1 (1–3)			
Lens awareness (0–10, 1)	1N	165	1 (1–2)	1 (1–2)	0.139	0.471	0.858
	2W	153	1 (1–4)	1 (1–4)			
	1M	144	1 (1–3)	1 (1–3)			
	3M	128	1 (1–3)	1 (1–3)			

* 1N = 1 night of lens wear, 2W = 2 weeks of lens wear, 1M = 1 month on lens wear, 3M = 3 months of lens wear.

## Data Availability

Data are available upon request from the corresponding author.

## Supplementary Materials

The following supporting information can be downloaded at: https://www.mdpi.com/article/10.3390/antibiotics11010058/s1, Table S1: Modified CLDEQ-8 questionnaire for monocular lens wear, Table S2: Ocular and subjective responses at baseline visit prior to lens wear and at the 4M study visit, one month after cessation of contact lens wear, Table S3: Ocular and subjective responses at dropouts and completed visits, Table S4: Lens surface characteristics and fit characteristics of Mel4 and control contact lenses at dropouts and completed visits.
