# Supplementary material for: Biocompatibility and Comfort during Extended Wear of Mel4 Peptide-Coated Antimicrobial Contact Lenses
Source: Antibiotics (Basel). 2022 Jan 3;11(1):58. doi: 10.3390/antibiotics11010058 (PMC8772808; doi:10.3390/antibiotics11010058)
Supplement: Supplementary file 1 [file antibiotics-11-00058-s001.zip › Supplementary tables.pdf]

Table S1: Modified CLDEQ-8 questionnaire for monocular lens wear

1	Questions about **EYE DISCOMFORT**						
	a) During a typical day in the past 2 weeks, how often did your eyes feel discomfort while wearing your contact lenses?						
	Right Eye:	☐ 0, Never	☐ 1, Rarely	☐ 2, Sometimes	☐ 3, Frequently	☐ 4, Constantly	
	Left Eye	☐ 0, Never	☐ 1, Rarely	☐ 2, Sometimes	☐ 3, Frequently	☐ 4, Constantly	
	b) When your eyes felt discomfort with your contact lenses, how intense was this feeling of discomfort…<br>At the end of the day?						
	Never Had it		Not at All Intense		Very Intense		
	Right Eye:	☐ 0	☐ 1	☐ 2	☐ 3	☐ 4	☐ 5
	Left Eye	☐ 0	☐ 1	☐ 2	☐ 3	☐ 4	☐ 5
2	Questions about **EYE DRYNESS**:						
	a) During a typical day in the past 2 weeks, how often did your eye feel dry?						
	Right Eye:	☐ 0, Never	☐ 1, Rarely	☐ 2, Sometimes	☐ 3, Frequently	☐ 4, Constantly	
	Left Eye	☐ 0, Never	☐ 1, Rarely	☐ 2, Sometimes	☐ 3, Frequently	☐ 4, Constantly	
	b) When your eyes felt dry, how intense was this feeling of dryness…At the end of the day?						
	Never Had it		Not at All Intense		Very Intense		
	Right Eye:	☐ 0	☐ 1	☐ 2	☐ 3	☐ 4	☐ 5
	Left Eye	☐ 0	☐ 1	☐ 2	☐ 3	☐ 4	☐ 5
3	Questions about **CHANGEABLE, BLURRY VISION**:						
	a) During a typical day in the past 2 weeks, how often did your vision change between clear and burry or foggy while wearing your contact lenses?						
	Right Eye:	☐ 0, Never	☐ 1, Rarely	☐ 2, Sometimes	☐ 3, Frequently	☐ 4, Constantly	
	Left Eye	☐ 0, Never	☐ 1, Rarely	☐ 2, Sometimes	☐ 3, Frequently	☐ 4, Constantly	
	b) When your vision was blurry, how noticeable was the changeable, blurry, or foggy vision … At the end of the day?						
	Never Had it		Not at All Intense		Very Intense		
	Right Eye:	☐ 0	☐ 1	☐ 2	☐ 3	☐ 4	☐ 5
	Left Eye	☐ 0	☐ 1	☐ 2	☐ 3	☐ 4	☐ 5
4	Questions about **CLOSING YOUR EYES**:						

	During a typical day in the past 2 weeks, how often did your eyes bother you so much that you wanted to close them?						
	Right Eye:	☐ 0, Never	☐ 1, Rarely	☐ 2, Sometimes	☐ 3, Frequently	☐ 4, Constantly	
	Left Eye	☐ 0, Never	☐ 1, Rarely	☐ 2, Sometimes	☐ 3, Frequently	☐ 4, Constantly	
5	Questions about **REMOVING YOUR LENSES**						
	How often during the past 2 weeks, did your eyes *bother you so much* while wearing your contact lenses that you felt as if you needed to stop whatever you were doing and take out your contact lenses?						
	Right Eye:	☐ 1, Never	☐ 2, Less than once a week	☐ 3, Weekly	☐ 4, Several times a week	☐ 5,Daily	☐ 6, Several times a day
	Left Eye	☐ 1, Never	☐ 2, Less than once a week	☐ 3, Weekly	☐ 4, Several times a week	☐ 5,Daily	☐ 6, Several times a day

Table S2: Ocular and subjective responses at baseline visit prior to lens wear and at the 4M study visit, one month after cessation of contact lens wear.

Variables	Visits with Lens	Sample (n)	Mel4 Lens	Control Lens	Linear Mixed Model		
			(Mean±SD)/ Median & Range	(Mean±SD)/ Median & Range	Lens (Mel4 vs. Control)	Visit	Lens vs Visit
**Conjunctival responses**							
**Bulbar Redness (0-4,0.1)**	**BL**	208	1.3±0.2	1.3±0.2	1.000	**0.001**	1.000
	**4M**	129	1.5±0.2	1.5±0.2			
**Limbal Redness (0-4,0.1)**	**BL**	208	1.1±0.3	1.1±0.3	0.440	**0.001**	0.438
	**4M**	129	1.3±0.2	1.3±0.2			
**Palpebral Redness (0-4,0.1)**	**BL**	208	1.4±0.3	1.4±0.3	0.769	**0.001**	0.670
	**4M**	129	1.5±0.2	1.5±0.2			
**Palpebral Roughness (0-4,0.1)**	**BL**	208	1.2±0.3	1.2±0.3	0.295	**0.001**	0.975
	**4M**	129	1.3±0.2	1.3±0.2			
**Corneal responses**							
**Centre (Extent; 0-4,1)**	**BL**	208	0 (0-0)	0 (0-0)	0.135	0.180	0.052
	**4M**	126*	0 (0-1)	0 (0-0)			
**Centre (Depth; 0-4,1)**	**BL**	208	0 (0-0)	0 (0-0)	0.135	0.180	0.052
	**4M**	126*	0 (0-1)	0 (0-0)			
**Centre (Type; 0-4,0.5)**	**BL**	208	0 (0-0)	0 (0-0)	0.135	0.180	0.052
	**4M**	126*	0 (0-2)	0 (0-0)			
**Nasal (Extent; 0-4,1)**	**BL**	208	0 (0-1)	0 (0-1)	0.577	0.205	0.056
	**4M**	126*	0 (0-1)	0 (0-0)			
**Nasal (Depth; 0-4,1)**	**BL**	208	0 (0-1)	0 (0-1)	0.577	0.205	0.056
	**4M**	126*	0 (0-1)	0 (0-0)			
**Nasal (Type; 0-4,0.5)**	**BL**	208	0 (0-1)	0 (0-1)	0.728	0.135	0.066
	**4M**	126*	0 (0-1)	0 (0-0)			
**Temporal (Extent; 0-4,1)**	**BL**	208	0 (0-1)	0 (0-1)	0.345	0.529	0.234
	**4M**	126*	0 (0-1)	0 (0-1)			

**Temporal (Depth; 0-4,1)**	**BL**	208	0 (0-1)	0 (0-1)	0.345	0.529	0.234
	**4M**	126*	0 (0-1)	0 (0-1)			
**Temporal (Type; 0-4,0.5)**	**BL**	208	0 (0-1)	0 (0-1)	0.161	0.647	0.161
	**4M**	126*	0 (0-1)	0 (0-0.5)			
**Superior (Extent; 0-4,1)**	**BL**	208	0 (0-1)	0 (0-0)	0.577	0.807	0.076
	**4M**	126*	0 (0-0)	0 (0-1)			
**Superior (Depth; 0-4,1)**	**BL**	208	0 (0-1)	0 (0-0)	0.577	0.807	0.076
	**4M**	126*	0 (0-0)	0 (0-1)			
**Superior (Type; 0-4,0.5)**	**BL**	208	0 (0-1)	0 (0-0)	0.577	0.807	0.076
	**4M**	126*	0 (0-0)	0 (0-1)			
**Inferior (Extent; 0-4,1)**	**BL**	208	0 (0-1)	0 (0-1)	0.872	**0.016**	0.514
	**4M**	126*	0 (0-1)	0 (0-1)			
**Inferior (Depth; 0-4,1)**	**BL**	208	0 (0-1)	0 (0-1)	0.698	**0.013**	0.697
	**4M**	126*	0 (0-1)	0 (0-1)			
**Inferior (Type; 0-4,0.5)**	**BL**	208	0 (0-1)	0 (0-1)	0.829	**0.012**	0.289
	**4M**	126*	0 (0-1)	0 (0-1)			
**Subjective responses**							
**Overall comfort (1-100,1)**	**BL**	208	97±4	97±4	0.671	**0.001**	0.671
	**4M**	129	94±4	94±5			
**Overall dryness (1-100,1)**	**BL**	208	4±8	4±8	0.989	0.082	0.659
	**4M**	129	6±4	6±4			

** Total no. = 129, missed data for three subjects.*

Table S3: Ocular and subjective responses at dropouts and completed visits.

Variables (range, incremental steps)	Visits with Lens	Dropouts			Completed visit		
		No of dropouts (n)	Mel4 Lens	Control Lens	Mel4 Lens	Control Lens	Total sample (n)
			(Mean±SD)/ Median & Range	(Mean±SD)/ Median & Range	(Mean±SD)/ Median & Range	(Mean±SD)/ Median & Range	
**Conjunctival responses**							
**Bulbar Redness (0-4,0.1)**	**2W**	4	1.6±0.3	1.6±0.3	1.5±0.2	1.5±0.2	153
	**1M**	2	1.4±0.1	1.4±0.1	1.6±0.2	1.6±0.2	144
	**3M**	2	1.7±0.0	1.7±0.1	1.6±0.2	1.6±0.2	129
**Limbal Redness (0-4,0.1)**	**2W**	4	1.3±0.2	1.3±0.2	1.2±0.2	1.2±0.2	153
	**1M**	2	1.2±0.1	1.2±0.1	1.3±0.3	1.3±0.3	144
	**3M**	2	1.5±0.1	1.5±0.1	1.4±0.2	1.4±0.2	129
**Palpebral Redness (0-4,0.1)**	**2W**	4	1.8±0.1	1.8±0.1	1.6±0.3	1.6±0.3	153
	**1M**	2	1.7±0.2	1.7±0.2	1.6±0.3	1.6±0.3	144
	**3M**	2	1.8±0.4	1.7±0.2	1.7±0.3	1.7±0.3	129
**Palpebral Roughness (0-4,0.1)**	**2W**	4	1.3±0.2	1.4±0.2	1.3±0.3	1.3±0.3	153
	**1M**	2	1.3±0.1	1.3±0.1	1.3±0.3	1.3±0.3	144
	**3M**	2	1.2±0.3	1.2±0.3	1.4±0.3	1.4±0.3	129
**Corneal responses**							
**Centre (Extent; 0-4,1)**	**2W**	4	0 (0-0)	0 (0-0)	0 (0-1)	0 (0-1)	153
	**1M**	2	0 (0-0)	0 (0-0)	0 (0-0)	0 (0-1)	144
	**3M**	2	0 (0-0)	0 (0-0)	0 (0-1)	0 (0-1)	129
**Centre (Depth; 0-4,1)**	**2W**	4	0 (0-0)	0 (0-0)	0 (0-1)	0 (0-1)	153
	**1M**	2	0 (0-0)	0 (0-0)	0 (0-0)	0 (0-1)	144
	**3M**	2	0 (0-0)	0 (0-0)	0 (0-1)	0 (0-1)	129
**Centre (Type; 0-4,0.5)**	**2W**	4	0 (0-0)	0 (0-0)	0 (0-1)	0 (0-1)	153
	**1M**	2	0 (0-0)	0 (0-0)	0 (0-0)	0 (0-1)	144
	**3M**	2	0 (0-0)	0 (0-0)	0 (0-1)	0 (0-1)	129
**Nasal (Extent; 0-4,1)**	**2W**	4	0 (0-0)	0 (0-0)	0 (0-1)	0 (0-1)	153
	**1M**	2	0 (0-0)	0 (0-0)	0 (0-1)	0 (0-2)	144
	**3M**	2	0 (0-0)	0 (0-0)	0 (0-0)	0 (0-1)	129
**Nasal (Depth; 0-4,1)**	**2W**	4	0 (0-0)	0 (0-0)	0 (0-1)	0 (0-1)	153
	**1M**	2	0 (0-0)	0 (0-0)	0 (0-1)	0 (0-2)	144
	**3M**	2	0 (0-0)	0 (0-0)	0 (0-0)	0 (0-1)	129
**Nasal (Type; 0-4,0.5)**	**2W**	4	0 (0-0)	0 (0-0)	0 (0-1)	0 (0-1)	153
	**1M**	2	0 (0-0)	0 (0-0)	0 (0-1)	0 (0-2)	144
	**3M**	2	0 (0-0)	0 (0-0)	0 (0-0)	0 (0-1)	129
**Temporal (Extent; 0-4,1)**	**2W**	4	0 (0-0)	0 (0-0)	0 (0-1)	0 (0-1)	153
	**1M**	2	0 (0-0)	0 (0-0)	0 (0-0)	0 (0-1)	144
	**3M**	2	0 (0-0)	0 (0-0)	0 (0-1)	0 (0-0)	129

**Temporal (Depth; 0-4,1)**	**2W**	4	0 (0-0)	0 (0-0)	0 (0-1)	0 (0-1)	153
	**1M**	2	0 (0-0)	0 (0-0)	0 (0-0)	0 (0-1)	144
	**3M**	2	0 (0-0)	0 (0-0)	0 (0-1)	0 (0-0)	129
**Temporal (Type; 0-4,0.5)**	**2W**	4	0 (0-0)	0 (0-0)	0 (0-1)	0 (0-1)	153
	**1M**	2	0 (0-0)	0 (0-0)	0 (0-0)	0 (0-0.5)	144
	**3M**	2	0 (0-0)	0 (0-0)	0 (0-1)	0 (0-0)	129
**Superior (Extent; 0-4,1)**	**2W**	4	0 (0-0)	0 (0-0)	0 (0-1)	0 (0-1)	153
	**1M**	2	0 (0-0)	0 (0-0)	0 (0-1)	0 (0-2)	144
	**3M**	2	0 (0-0)	0 (0-0)	0 (0-1)	0 (0-1)	129
**Superior (Depth; 0-4,1)**	**2W**	4	0 (0-0)	0 (0-0)	0 (0-1)	0 (0-1)	153
	**1M**	2	0 (0-0)	0 (0-0)	0 (0-1)	0 (0-2)	144
	**3M**	2	0 (0-0)	0 (0-0)	0 (0-1)	0 (0-1)	129
**Superior (Type; 0-4,0.5)**	**2W**	4	0 (0-0)	0 (0-0)	0 (0-1)	0 (0-0.5)	153
	**1M**	2	0 (0-0)	0 (0-0)	0 (0-1)	0 (0-1.5)	144
	**3M**	2	0 (0-0)	0 (0-0)	0 (0-1)	0 (0-1)	129
**Inferior (Extent; 0-4,1)**	**2W**	4	0 (0-0)	0 (0-1)	0 (0-1)	0 (0-2)	153
	**1M**	2	1 (0-1)	1 (0-1)	0 (0-1)	0 (0-2)	144
	**3M**	2	0 (0-0)	0 (0-0)	0 (0-2)	0 (0-2)	129
**Inferior (Depth; 0-4,1)**	**2W**	4	0 (0-0)	0 (0-1)	0 (0-1)	0 (0-2)	153
	**1M**	2	1 (0-1)	1 (0-1)	0 (0-1)	0 (0-2)	144
	**3M**	2	0 (0-0)	0 (0-0)	0 (0-2)	0 (0-2)	129
**Inferior (Type; 0-4,0.5)**	**2W**	4	0 (0-0)	0 (0-1)	0 (0-1)	0 (0-2)	153
	**1M**	2	1.5 (1-2)	0.5 (0-1)	0 (0-1)	0 (0-2)	144
	**3M**	2	0 (0-0)	0 (0-0)	0 (0-2)	0 (0-2)	129
**Subjective responses**							
**Overall comfort (1-100,1)**	**2W**	4	96±5	96±5	91±7	91±8	153
	**1M**	2	60±42	60±42	90±7	91±9	144
	**3M**	2	93±4	93±4	88±8	89±7	129
**Overall dryness (1-100,1)**	**2W**	4	4±4	4±4	11±11	11±11	153
	**1M**	2	45±49	45±49	11±11	12±12	144
	**3M**	2	8±4	8±4	13±11	13±11	129
**Edge awareness (0-10,1)**	**2W**	4	1 (1-2)	1 (1-1)	1 (1-4)	1 (1-5)	153
	**1M**	2	1 (1-1)	1 (1-1)	1 (1-3)	1 (1-3)	144
	**3M**	2	1 (1-1)	1 (1-1)	1 (1-3)	1 (1-3)	129
**Lens awareness (0-10,1)**	**2W**	4	1 (1-1)	1 (1-1)	1 (1-4)	1 (1-4)	153
	**1M**	2	1 (1-1)	1 (1-1)	1 (1-3)	1 (1-3)	144
	**3M**	2	1 (1-1)	1 (1-1)	1 (1-3)	1 (1-3)	129

Table S4: Lens surface characteristics and fit characteristics of Mel4 and control contact lenses at dropouts and completed visits.

Variables (range, incremental steps)	Visits with Lens	Dropouts			Completed visit		
		No of dropouts (n)	Mel4 Lens (Mean±SD) / Median & Range	Control Lens (Mean±SD)/ Median & Range	Mel4 Lens (Mean±SD)/ Median & Range	Control Lens (Mean±SD)/ Median & Range	Total sample (n)
**Lens surface characteristics**							
**Front surface wetting (0-4,0.1)**	**2W**	4	3.7±0.1	3.7±0.1	3.6±0.3	3.6±0.3	153
	**1M**	2	3.5±0.1	3.5±0.0	3.6±0.3	3.5±0.3	144
	**3M**	2	3.4±0.6	3.4±0.6	3.6±0.3	3.5±0.3	129
**Front surface deposits (0-4,0.1)**	**2W**	4	0.2±0.2	0.2±0.2	0.6±0.5	0.6±0.5	153
	**1M**	2	0.8±0.4	0.8±0.4	0.6±0.6	0.6±0.6	144
	**3M**	2	1.0±0.7	1.0±0.7	0.7±0.6	0.7±0.6	129
**Back surface debris (0-4,0.1)**	**2W**	4	0.2±0.2	0.2±0.2	0.3±0.4	0.3±0.4	153
	**1M**	2	0.8±0.4	0.8±0.4	0.4±0.5	0.4±0.5	144
	**3M**	2	1.0±0.7	1.0±0.7	0.4±0.5	0.4±0.5	129
**Lens fit characteristics**							
**Centration X-axis (-1to+1, 0.1 mm)**	**2W**	4	0 (0.0 - 0.3)	0 (0.0 - 0.3)	0 (-0.3 - 0.2)	0 (-0.3 - 0.2)	153
	**1M**	2	0 (0 - 0)	0 (0 - 0)	0 (-0.5 - 0.2)	0 (-0.5 - 0.0)	144
	**3M**	2	0 (0 - 0)	0 (0 - 0)	0 (-0.3 - 0.2)	0 (-0.3 - 0.2)	129
**Centration Y-axis (-1to+1, 0.1 mm)**	**2W**	4	0.1 (-0.3 - 0.3)	0.1 (-0.3 - 0.3)	0 (-0.3 - 0.5)	0 (-0.3 - 0.5)	153
	**1M**	2	0 (0 - 0)	0 (0 - 0)	0 (-0.4 - 0.4)	0 (-0.3 - 0.4)	144
	**3M**	2	0 (0 - 0)	0 (0 - 0)	0 (-0.2 - 0.3)	0 (-0.2 - 0.3)	129
**Primary gaze movement (0-10,0.1)**	**2W**	4	0.4±0.1	0.3±0.1	0.4±0.1	0.4±0.1	153
	**1M**	2	0.3±0.0	0.3±0.0	0.4±0.1	0.4±0.1	144
	**3M**	2	0.4±0.0	0.4±0.0	0.4±0.1	0.4±0.1	129
**Primary gaze lag (0-10,0.1)**	**2W**	4	0.1±0.1	0.1±0.1	0.1±0.1	0.1±0.1	153
	**1M**	2	0.2±0.1	0.2±0.1	0.2±0.1	0.2±0.1	144
	**3M**	2	0.2±0.0	0.2±0.0	0.2±0.1	0.2±0.1	129
**Tightness (0-100,1) %**	**2W**	4	41±3	41±3	42±3	42±3	153
	**1M**	2	40±0	40±0	41±3	41±3	144
	**3M**	2	40±0	40±0	42±3	42±3	129
**Overall Acceptance (0-4,0.1)**	**2W**	4	3.7±0.2	3.7±0.2	3.8±0.1	3.8±0.1	153
	**1M**	2	3.9±0.1	3.9±0.1	3.8±0.1	3.8±0.1	144
	**3M**	2	3.8±0.0	3.8±0.0	3.8±0.1	3.8±0.1	129
